# The costs of disability in Australia: a hybrid panel-data examination

**DOI:** 10.1186/s13561-020-00264-1

**Published:** 2020-03-14

**Authors:** Binh Vu, Rasheda Khanam, Maisha Rahman, Son Nghiem

**Affiliations:** 1 University of Southern Queensland, Toowoomba, Australia; 2 Institute of Research and Development, Duy Tan University, Da Nang, 550000 Vietnam; 3 Royal Brisbane & Women’s Hospital, Brisbane, Australia; 4 Centre for Applied Health Economics, 170 Kessels Road Sir Samuel Griffith Centre (N78) 1.11, Queensland, Nathan QLD 4111 Australia

**Keywords:** Cost of disability, Standard of living, Panel data, Hybrid estimator, Australia

## Abstract

**Background:**

Over four million people in Australia have some form of disability, of whom 2.1 million are of working age. This paper estimates the costs of disability in Australia using the standard-of-living approach. This approach defines the cost of disability as additional income required for people with a disability to achieve a similar living standard to those without a disability. We analyse data from the Household, Income and Labour Dynamics in Australia (HILDA) Survey using a hybrid panel data model. To the best of our knowledge, this is the first study to examine the costs of disability in Australia using a high quality, large, nationally-representative longitudinal data set.

**Methods:**

This study estimates the costs of disability in Australia by using the Standard of Living (SoL) and a dynamic model approach. It examines the dynamics of disability and income by using lagged disability and income status. The study also controls for unobserved individual heterogeneity and endogeneity of income. The longitudinal specification in this study allows us to separate short- and long-run costs of disability using a hybrid panel data regression approach.

**Results:**

Our results show that people with a disability need to increase their adult-equivalent disposable income by 50% (in the short-run) to achieve the same standard of living as those without a disability. This figure varies considerably according to the severity of the disability, ranging from 19% for people without work-related limitations to 102% for people with severe limitations. Further, the average cost of disability in the long-run is higher and it is 63% of the adult-equivalent disposable income.

**Conclusions:**

Firstly, our results show that with the same level of income, the living standard is lower in households with people with a disability compared to households without members with a disability. This indicates a strong relationship between poverty and disability. However, current poverty measures do not take into account disability, therefore, they fail to consider substantial differences in poverty rates between people with and without a disability. Secondly, the estimated costs reflected in this study do consider foregone income due to disability. Therefore, policymakers should seriously consider adopting disability-adjusted poverty and inequality measurements. Thirdly, increasing the income (e.g. through government payments) or providing subsidised services for people with a disability may increase their financial satisfaction, leading to an improved living standard. The results of this study can serve as a baseline for the evaluation of the National Disability Insurance Scheme (NDIS).

## Introduction

About four million people in Australia have some form of disability, of whom 2.1 million are of working age [1]. There is a consensus that people with a disability need additional income to achieve a similar living standard to those without a disability. An Australian study by [2] showed that the costs of disability in households with at least one family member with a disability was 37% of the disposable income (i.e. people with a disability need to increase disposable income by 37% to have the same living standard as those without a disability). However, the cost estimated by [2] is based on an outdated data set: the 1998-1999 Household Expenditure Survey. Further, [2]’s cross-sectional study was unable to control for potential confounders. In addition, since 2007, Australia’s current health landscape has changed significantly and the nation is currently undergoing a major reform in disability care with the introduction of the National Disability Insurance Scheme (NDIS). The NDIS aims to support all Australians below 65 years of age with a permanent and significant disability to achieve greater independence, community involvement, employment and improved wellbeing [3]. The scheme was piloted in several trial sites around Australia from July 2013 and was rolled out gradually to the rest of Australia from July 2016. It will be in full operation in 2020. Thus, there is an urgent need to estimate the costs of disability in Australia using a contemporary data set. We fill this gap in knowledge by estimating the costs of disability in Australia using recent longitudinal data (2001-2016) from the Household, Income and Labour Dynamics in Australia (HILDA) Survey.

We contributed to the existing literature in the following ways. Firstly, we provided up-to-date estimates of the costs of disability in Australia using a large, nationally-representative longitudinal data set. Secondly, use of longitudinal data allowed us to control for confounders such as previous disability and income status which affect current disability and thus current living standard and disability costs. The added benefit of using previous disability and income status was the opportunity to examine long-run costs of disability, which was not possible in cross-sectional studies (e.g.,[4]; [2]). Thus we were able to distinguish between short-run (contemporaneous) and long-run (lagged) costs of disability. Finally, we were able to control for individual-specific unobserved effects by using a hybrid panel regression model, which mitigated the bias caused by unobserved effects. Results from a base model showed that people with a disability need to increase adult-equivalent disposable income by 50% to achieve a similar living standard as those without a disability. However, the cost varied with the level of functional limitations caused by the disability, ranging from 19% for those with no work-related limitations to 102% for those with severe limitations. Note that the cost of disability in this study was estimated implicitly rather than explicitly, similar to most cost-of-illness studies. Thus, indirect costs such as loss of productivity due to disability were also implicitly included in the additional income required to make the standard of living of people with a disability similar to that of those without a disability.

## Literature review

There are several approaches to measure the costs of disability, and each approach has its own advantages and disadvantages [5, 6]. One approach uses the receipt of a disability payment as a proxy for the costs of disability. An implicit assumption in this approach is that disability payments perfectly represent disability costs, which can be questionable as there may be other hidden costs which cannot be represented through receipts. The other approach is based on expert opinions on the costs of disability. The main difficulty with this approach is that disability is a complex concept and cost estimates from experts or people with a disability may vary considerably. Revealed preference is the third approach to estimate the costs of disability. This involves estimating the consumption pattern of people with a disability and matching that of individuals without a disability. However, this approach is based on the assumption that both groups were given alternatives to make their consumption decisions. This assumption may not hold in practice as people with a disability often face comparatively fewer choices. The final approach is referred to as the “standard of living (SoL)” approach. This consists of indirectly estimating the disability costs as the amount of additional income needed to make the living standard of people with a disability similar to that of people without a disability. We use the SoL approach because of its relevance to the available data and its increasing popularity in the literature (for a recent review, see [7]).

We focus on reviewing the most relevant studies using the SoL approach to estimate the costs of disability within developed countries. A study by [4] was one of the earliest studies deploying this approach to estimate disability costs in the United Kingdom using the 1996/1997 Family Resources Survey (FRS) and the British Household Panel Survey (BHPS). They found that the extra costs of disability varied considerably with the data sets, choice of living standard indicators, and household structure. For example, the cost of disability for households that had members with a disability was 14% of mean income when analysing FRS data with a dummy variable capturing whether the household had “any savings” used as a proxy for standard of living. The estimate increased to 50% when the BHPS data were analysed, and a categorical variable on the self-reported “financial situation” of the household was selected to represent the living standard. Morciano et al. [6] updated these analyses to the 2007/2008 wave of the FRS data and took into account the latent nature of disability and living standard. They focused on estimating the costs of disability among people over the pension age (65 for men and 60 for women) in households with a single person or a couple. They used a series of variables to construct the indicators for living standard (e.g. ability to repair or keep the home in decent conditions, affordability of holidays, hobbies and leisure activities) and different indicators of disability (e.g. difficulties with mobility, communication and memory). Their results showed that disability costs predicted by linear, log-linear and log-quadratic models were 55%, 65% and 62% of net weekly income, respectively.

Cullinan et al. [8] examined both the short-run and long-run economic costs of disability using the Irish survey data of the 1995–2001 period. They found that for people with a severe level of disability, the short-run costs (30% of weekly income) were higher than the long-run costs (23.6%). However, for those with a lower level of disability the short-run costs (17.5%) were lower than the long-run costs (20.3%). Both the short- and long-run disability costs became statistically insignificant when controlling for unobservable characteristics. Anton et al. [9] compared the cost of disability between 31 EU countries using the SOL approach, with living standard indicators being “subjective well-being” and “asset ownership”. They found strong positive correlations between disability costs and GDP per capita. Their estimated disability costs ranged from 17% to 99% for subjective wellbeing and from 16% to 155% for asset ownership.

In Australia, quantitative research on disability costs is limited. The only available Australian study [2] found that households with at least one family member with a disability need an increase of disposable income by 37% to achieve a similar level of living standard to those families living without a disability. He also found that the income gap increased with the level of disability, reaching 40% to 49% of income for those with a severe restriction. However, the cross-sectional nature of the data set used in the study did not allow for examining transient effects or controlling of unobserved individual-specific characteristics.

In summary, although numerous studies have investigated the cost of disability in developed countries, no previous study has used nationally representative longitudinal data to estimate the costs of disability in Australia. In addition, the only available Australian study [2] is now out-dated. Therefore, the current study will add significantly to the existing literature. Further, a more precise and detailed estimate of the costs of disability in Australia in the present period will provide a critical baseline for accurate future evaluations of the National Disability Insurance Scheme (NDIS), which will be implemented fully in 2020.

## Methods

**Fig. 1:**
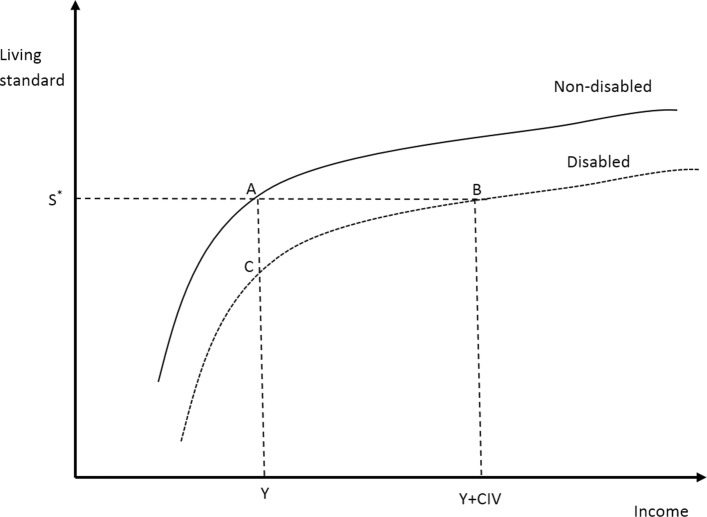
The relationship between living standard, income and disability

The costs of disability in this study are calculated using the SoL and a dynamic model approach, which is similar to [8]. SoL estimates the additional income required by people with disability to have a similar living standard as people without a disability. People with a disability have a lower living standard at the same level of income or require higher income to maintain the same living standard as those without a disability, if all other factors remain constant. This is because physical and mental disabilities often result in lower productive capabilities, resulting in a poorer ability to work to gain income or a narrower range of potential occupations. Also, having disability incurs costs associated with medication, functional adaptation and health care. As illustrated in Fig. 1, for any given income *Y*, the living standard of people with a disability (point *C*) is lower than that of people without a disability (point *A*). To maintain the same standard of living (*S*^∗^) as people with no disability, an additional amount of income (i.e. the “compensating income variation” – *CIV*) is needed to shift the position of people with a disability from point *C* to point *B*.

Empirically, the costs of disability using the standard of living approach is specified as

1 $$ S_{it}=\beta_{0}+\beta_{1}Y_{it}+\beta_{2}D_{it}+\gamma X_{it}+(\alpha_{i}+\epsilon_{it})  $$

where *S*_*it*_ represents the standard of living of individual *i* at time period *t*; *Y* is the logarithm of inflation-adjusted disposable income per adult-equivalent; *D* is the disability status, *X* is a vector of individual, household and neighbourhood characteristics; and the composite error term consists of individual-specific unobserved characteristics (*α*_*i*_) and random noise (*ε*_*it*_).

The additional amount of income (i.e., the “compensating income variation” – CIV) needed to keep the living standard of people with a disability (*S*_(*CIV*+*Y*,*D*=1)_) equal to that of people without a disability (*S*_(*Y*,*D*=0)_) can be estimated by replacing their respective values of income and disability status into Eq. (1):

2 $$ \begin{array}{cc} (CIV+Y)\times\beta_{1}+\beta_{2} & =Y\times\beta_{1}\end{array}  $$

and thus, the percentage income gap due to disability is $\frac {-\beta _{2}}{\beta _{1}}$.

Due to the presence of individual-specific unobserved characteristics *α*_*i*_, the composite error term may be correlated with other observable covariates. Thus, applying standard regression to Eq. (1) may produce biased estimates. A random-effect estimator assumes that individual-specific unobserved characteristics (*α*_*i*_) follow a normal distribution with zero mean and non-zero variance, and, critically, are uncorrelated with observable covariates. Alternatively, a fixed-effect estimator eliminates the time-invariant unobserved individual-specific characteristics *α*_*i*_ by taking the mean difference of the outcome and covariates, as follows:

3 $$ {}S_{it}-\bar{S_{i}}=\beta_{w}([X_{it}-\bar{X_{i}}]+[Y_{it}-\bar{Y_{i}}]+[D_{it}-\bar{D_{i}}])+(\epsilon_{it}-\bar{\epsilon_{i}})  $$

where *β*_*w*_ give the within (or fixed) effects of the covariates on the outcome variables (i.e. how within-individual variations in covariates affect within-individual changes in the outcome). However, this approach cannot yield estimates of the effects of observable time-invariant characteristics such as gender and ethnicity. For categorical outcomes, a fixed-effect estimator will eliminate all observations from individuals who report the same standard of living over time.

From Eq. (3), *S*_*it*_ can be expressed as

4 $$ \begin{array}{ll} S_{it}&= \bar{S_{i}}+\beta_{w}([X_{it}-\bar{X_{i}}]+[Y_{it}-\bar{Y_{i}}]+[D_{it}-\bar{D_{i}}])\\&\quad+(\epsilon_{it}-\bar{\epsilon_{i}})\\ &=\beta_{w}(\bar{X_{i}}+\bar{Y_{i}}+\bar{D_{i}})+\bar{\alpha_{i}}+\bar{\epsilon_{i}}+\beta_{w}([X_{it}-\bar{X_{i}}]\\&\quad+[Y_{it}-\bar{Y_{i}}]+[D_{it}-\bar{D_{i}}])+(\epsilon_{it}-\bar{\epsilon_{i}})\\ &= +\beta_{w}([X_{it}-\bar{X_{i}}]+[Y_{it}-\bar{Y_{i}}]+[D_{it}-\bar{D_{i}}])\\&\quad+\beta_{w}(\bar{X_{i}}+\bar{Y_{i}}+\bar{D_{i}})+\bar{\alpha_{i}}+\epsilon_{it} \end{array}  $$

Mundlak [10] proposed a correlated random-effect estimator where the time-invariant individual unobserved characteristics (*α*_*i*_) are allowed to be correlated with the time-average of potentially endogenous observable covariates:

5 $$ \alpha_{i}=\gamma(\bar{X_{i}}+\bar{Y_{i}}+\bar{D_{i}})+\varepsilon_{i}  $$

where *ε*_*i*_ is random noise. Since the individual unobserved effects are time-invariant ($\alpha _{i}=\bar {\alpha _{i}}$) and hence the value of *α*_*i*_ in Eq. 5 can be used to replace $\bar {\alpha _{i}}$ in Eq. 4 to obtain hybrid estimator proposed by [11]:

6 $$ {}{\begin{aligned}S_{it}=\beta_{w}([X_{it}-\bar{X_{i}}]+[Y_{it}-\bar{Y_{i}}]+[D_{it}-\bar{D_{i}}])+\beta_{b}(\bar{X_{i}}+\bar{Y_{i}}+\bar{D_{i}})+\epsilon_{it} \end{aligned}}  $$

where *β*_*b*_, which is a combination of $\bar {\beta }$ in Eq. 4 and *γ* in Eq. 5, represent between effects. Equation 6 can be estimated using an ordered logit random effects estimator, with the common indicator of standard of living as a ranking of financial satisfaction. This specification allows for the conducting of a Hausman-like test by using a Wald test for the equality of within- and between-effects parameters (*β*_*w*_=*β*_*b*_). This can be further tested using robust standard errors estimators, and it does not depend on the positive definiteness of covariance matrices [12]. The static specification in Eq. 6, however, does not reflect the fact that income and disability in the previous period can affect the standard of living in the current period. Thus, we also include the lagged value of disability status and income to specify this dynamic relationship. The advantage of this specification is that we are able to separate the contemporaneous disability costs (calculated using current period parameters) with the long-run costs (estimated using lagged parameters). Note that the outcome of interest in this study is the cost of disability or the ratio of $\frac {-\beta _{2}}{\beta _{1}}$ in Eq. 1. Thus, the term dynamic in this study refers to the inter-temporal relationship between disability status and income, rather than the inclusion of a lagged dependent variable in the model as a traditional dynamic specification.

## Data

### Data source and variable selection

The data used in this study come from the first 16 waves of the Household, Income and Labour Dynamics in Australia (HILDA) Survey– a nationally representative longitudinal study of Australian households. The annual survey began in 2001 and collected a wide range of information on relationships, child care, employment, income, health and wellbeing, from all household members aged 15 years and older [13]. The HILDA Survey applied a multi-stage sampling approach to select the sample. In the first stage, 488 Census Collection Districts, each consisting of about 200-250 households, were selected by State and metropolitan status. In the second stage, 22-23 dwellings were selected from each Census Collection District. Finally, up to three households were selected from each dwelling. The main method of data collection was through face-to-face interviews but a small proportion of telephone interviews were also conducted for members who moved to locations outside the areas covered by interviewers. The survey attained a reasonably high response rate of 66% at the household level and 61% at the individual level [14]. The HILDA Survey followed the progress of participating households over time by including new participants: those who were children of participating households and became 15 years of age; those who began sharing a residence with participating households; and those who were married to or had children with participating household members. The wave-on-wave retention rates in the HILDA Survey were remarkably high, of around 95%.

### Variable selection

#### Dependent Variable

There is a wide range of variables that have been selected as measures of the standard of living in the literature, including subjective wellbeing [15], and self-reported financial situation [6]. We chose financial satisfaction as an indicator of the living standard, rather than overall subjective wellbeing, to represent SoL. Financial satisfaction is a more appropriate indicator because if income is sufficient to support the additional needs of a person living with a disability, then their standard of living will be similar to that of a person living without a disability. On the other hand, subjective wellbeing is dependent on factors such as the psychosocial status of an individual and is thus an unreliable indicator of living standard. Furthermore, additional income may not be able to restore the subjective overall wellbeing of people with a disability, but it could help them to achieve a similar level of financial satisfaction to those without a disability. In addition, financial satisfaction as an indicator is practical because results based on this can be easily translated into measurable policy targets, such as the optimal amount of financial support needed for people with a disability. Thus, we selected financial satisfaction as the proxy for SoL, with a range from 0 for “totally dissatisfied” to 10 for “totally satisfied” as the proxy for SoL. As a sensitivity test, we also approximate living standards via a dummy variable capturing whether the household can mobilise $2,000-$3,000 from savings. One could argue that a different choice of savings level would better represent financial stability but data limitation does not allow us to explore this path further.

#### Independent variables

Disability status was measured through the question “Do you have any long-term health condition, impairment or disability that restricts you in your everyday activities, and has lasted or is likely to last, for 6 months or more?” To measure the severity of a disability, we also considered responses to the question: “Could you pick a number between 0 and 10 to indicate how much your condition[s] limit[s] the amount of work you can do? An answer of 0 means “not at all” and an answer of 10 means you are “unable to do any work”. For ease of interpretation, we recoded responses into three categories: no limitation (score of 0); moderate limitation (scores of 1-6); and severe limitation (scores of 7-10). Also, we have assumed “no limitation” for people who reported having a disability but their response to the severity of their disability question is missing. Model covariates included age, gender, ethnicity, education level and employment of the respondent, household size, household income, type of tenure, and region of residence. The annual income variable was adjusted for inflation using the consumer price index at 2016 prices. We converted income to adult-equivalent income using the modified OECD-equivalence scale, which allocates a coefficient of 1 to the first adult, 0.5 to each of the remaining adults and 0.3 to each child under 15 [16]. The sample size of our study included all individuals in the HILDA Survey with no missing data on the selected variables.

### Descriptive statistics

Table 1 shows the prevalence of disability with various levels of severity over time and the associated standard of living. On average, 27.3% of the survey individuals have a disability or long-term health condition, which is comparable with the Irish figure of 28% [8] but considerably higher than the 18.5% figure reported by the Survey of Disability, Ageing and Carers (SDAC) in 2009 [17]. One possible reason for the difference is that the definition of disability in this study is broader: “having any long-term health condition, impairment or disability”. However, the proportion of households that have members with a disability causing limitation was lower when the severity was taken into account. The percentage of households with members with a disability having a moderate limitation and a severe limitation to work were 11.6% and 6.1%, respectively, making a total of 17.7%. The remaining 9.6% are people with long-term health conditions or who report having a disability but face no limitation to work.

**Table 1:** Disability status and standard of living over time

	Disability prevalence (%)				Standard of living (range 0-10)				
Ave	Any	Severity (limitation to work)			No	Any	Severity (limitation to work)		
	disability	No	Moderate	Severe	Disability	disability	No	Moderate	Severe
1	23.7	7.0	11.3	5.3	6.3	5.7	6.1	5.6	5.3
2	22.2	6.0	10.6	5.7	6.2	5.7	6.1	5.8	5.2
3	28.0	9.0	13.0	6.0	6.5	6.1	6.5	6.0	5.6
4	26.5	9.2	11.2	6.0	6.6	6.0	6.4	5.9	5.6
5	28.2	9.9	11.9	6.4	6.6	6.1	6.5	6.0	5.6
6	26.8	9.5	11.5	5.8	6.5	6.1	6.4	6.0	5.6
7	27.2	9.5	11.6	6.1	6.7	6.3	6.7	6.2	5.7
8	25.9	8.7	11.1	6.1	6.7	6.1	6.6	6.1	5.5
9	28.6	11.1	11.5	6.1	6.6	6.1	6.4	6.0	5.7
10	27.0	10.0	10.9	6.2	6.5	6.1	6.3	6.0	5.8
11	27.5	10.1	11.3	6.1	6.6	6.1	6.4	6.0	5.6
12	27.2	10.2	11.1	6.0	6.7	6.1	6.5	6.1	5.4
13	30.2	12.0	11.8	6.4	6.7	6.2	6.5	6.1	5.7
14	28.8	10.5	12.0	6.4	6.7	6.2	6.5	6.2	5.6
15	28.8	10.3	12.0	6.5	6.8	6.2	6.5	6.2	5.8
16	27.7	9.3	11.9	6.5	6.8	6.2	6.6	6.2	5.7
Total	27.3	9.6	11.6	6.1	6.6	6.1	6.4	6.1	5.6

The standard of living (proxied by financial satisfaction) decreased with increasing disability severity levels. For example, the average living standard for people with a disability with no limitation, some limitation and severe limitation to work were 6.4, 6.1 and 5.6, respectively. However, the living standard of people without a disability was substantially higher than that of people with a disability. This pattern was consistent from Wave 1 to Wave 16. While there was no clear trend on the prevalence of disability, the living standard has improved slightly over time across all disability severity levels.

Table 2 shows significant differences between people with and without a disability in a range of variables used in the models, with the exception of the gender of household heads.

**Table 2:** Descriptive statistics

Selected variables	Mean	SD	No disability	Disability	Diff. (*p*-val.)
OECD-equivalent disposable income (2016 prices)	50,719	47,712	53,892	42,266	0.00
Overall life satisfaction (0-10)	7.92	1.48	8.05	7.56	0.00
Satisfaction with financial situation (0-10)	6.46	2.25	6.60	6.09	0.00
Can mobilise $2000-$3000 from saving (Yes=1)	0.70	0.46	0.71	0.68	0.00
Age (Years)	44	19	41	55	0.00
Gender of respondent (Female=1)	0.53	0.50	0.52	0.54	0.00
Aboriginal and Torres Strait Islanders	0.02	0.15	0.02	0.03	0.00
Housing=Owns/mortgage	0.70	0.46	0.70	0.68	0.00
Housing=Private renting	0.23	0.42	0.25	0.20	0.00
Housing=Social renting	0.05	0.21	0.03	0.09	0.00
Housing=Live there free	0.03	0.16	0.02	0.03	0.00
Employment=Employed	0.48	0.50	0.55	0.29	0.00
Employment=Unemployed	0.03	0.17	0.028	0.031	0.01
Employment=Not in labour force	0.13	0.34	0.09	0.23	0.00
Employment=FT student	0.10	0.30	0.11	0.04	0.00
Employment=Retired	0.16	0.37	0.10	0.35	0.00
Employment=Self-employed	0.10	0.30	0.11	0.07	0.00
Marriage=Married/cohabitating	0.63	0.48	0.64	0.59	0.00
Marriage=Divorced/separated	0.14	0.35	0.11	0.24	0.00
Marriage=Never married	0.23	0.42	0.26	0.17	0.00
Region=Major city	0.62	0.49	0.63	0.58	0.00
Region=Inner regional	0.36	0.48	0.35	0.41	0.00
Region=Outer/Remote	0.02	0.14	0.02	0.02	0.00
Education=Postgraduate	0.09	0.29	0.10	0.07	0.00
Education=University	0.13	0.34	0.14	0.09	0.00
Education=Diploma or certificate	0.29	0.45	0.29	0.29	0.04
Education=Year 12	0.15	0.36	0.16	0.11	0.00
Education=Below Year 12	0.34	0.47	0.31	0.44	0.00
Household size	2.94	1.48	3.08	2.51	0.00
SEIFA=1st quintile	0.20	0.40	0.18	0.27	0.00
SEIFA=2nd quintile	0.20	0.40	0.20	0.21	0.00
SEIFA=3rd quintile	0.19	0.39	0.20	0.18	0.00
SEIFA=4th quintile	0.20	0.40	0.21	0.18	0.00
SEIFA=5th quintile	0.20	0.40	0.22	0.15	0.00

People without a disability were better-off with an average adult-equivalent disposable income of $53,892 per year, which was 27.5% higher than the figure of $42,266 for people with a disability. On average, people with a disability lived in smaller households, were more likely to be of Aboriginal and Torres Strait Islander descent, had lower education attainment, were more likely to live in socially rented/subsidised properties, or lived in more disadvantaged areas, as proxied by quintiles of the Socio-Economic Index for Areas (SEIFA). People with a disability also had lower levels of satisfaction with life, and the magnitude of the difference was substantial (8.05 versus 7.56). Likewise, differences in financial satisfaction levels (6.6 versus 6.1) and the probability of being able to mobilise $2,000-$3,000 from savings (71% versus 68%) were substantial. This suggests that using the ’level of satisfaction with life’ as a proxy for the standard of living may result in higher estimates of the costs of disability.

## Results

We first estimated the costs of disability in a pooled model, where disability at all levels of severity (proxied by the limitation to work) was estimated together. The Hausman-like specification test rejected (*χ*^2^(4)=251, *p*-val=0.00) the null hypothesis that the between and within parameters are equal, suggesting that the within parameters were preferred and hence we focus on reporting and discussing results based on these parameters. Table 3 shows that the magnitude of the disability parameters (in absolute value), and the estimated disability costs, increase with the level of severity. For example, contemporary costs, estimated as the ratio of the disability and income parameters in the current period, increased from 19% (i.e., $\frac {0.09}{0.46}$) for those with no work-related limitation, to 71% for those with some limitation and 102% for those with severe limitation (Table 3). However, the long-term disability costs, estimated as the ratio of the lagged disability to the lagged income parameter, increased at a slower pace, from 37% for those with no limitations to 94% for those with severe limitations. As expected, the estimated cost of disability using the disability indicator that disregards the severity of limitations lies in the middle of estimate costs based on different severity levels.

**Table 3:** Costs of disability by severity: pooled model (dependent variable: satisfaction with the financial situation)

Key variables	Current period		Lagged period	
	Coef (SE)	Contemporaneous costs (% of income)	Coef (SE)	Long-termcosts (% of income)
*Disability severity*				
No limitation	-0.09*** (0.02)	19%	-0.07*** (0.02)	37%
Some limitation	-0.33*** (0.02)	71%	-0.12*** (0.02)	63%
Severe limitation	-0.47*** (0.03)	102%	-0.18*** (0.03)	94%
Log of Income	0.46*** (0.01)		0.19*** (0.01)	
Hausman test	*χ*^2^(4):	251	*p*-val:	0.00
*Any disability*				
Disability	-0.23*** (0.01)	50%	-0.12*** (0.01)	63%
Log of income	0.46*** (0.01)		0.19*** (0.01)	

Note: Other covariates include age, gender, ethnicity education level, marital status and employment status of the respondent; household size, housing tenure status, region of residence (rural vs urban), SEIFA quintile, and a time trend.

In particular, the contemporaneous and long-term costs of having a disability were 50% and 63% of adult-equivalent annual income, respectively. These estimates are higher than those reported by [2]. This difference is likely to emerge because Saunders applied a standard regression model that did not account for unobserved individual-specific characteristics. For comparison, we applied a standard ordered logit regression same as [2] to Eq. 1 (instead of a hybrid ordered logit estimator in Eq. 6) and found that the additional costs for households with people with a disability were 37% of their equivalised disposable income, which is the same as the findings of [2]. This comparison result suggests that the inability of applying a panel data analysis may underestimate the disability costs.

### Sensitivity test

As a sensitivity test, we used the saving capacity of the household as a proxy for the living standard. Findings were similar: the disability costs increased with the level of severity (Table 4). However, the magnitudes of the cost estimates were smaller than in the main models for financial satisfaction. For example, the contemporary estimates of the additional cost for people with a disability and no work-related limitation increased to 13% and the costs for those with severe work-related limitations increased to 71%. The long-run disability cost estimates using the lagged parameters were more substantial, ranging from 62% for those without a work-related limitation to 118% for those with severe limitations. Similarly, the cost estimates that disregard the severity of disability were 31% and 77% for the long-term, respectively. We also performed an analysis using the overall level of satisfaction with life as a proxy for standard of living. As we expected, the cost estimates using this approach were much higher (people with a disability need to increase their disposable income by 300% to have the same level of overall satisfaction as those without a disability).

**Table 4:** Costs of disability by severity: pooled model, using savings to represent the standard of living

Key variables	Current period		Lagged period	
	Coef (SE)	Contemporaneous costs (% of income)	Coef (SE)	Long-term Costs (% of income)
*Disability severity*				
No limitation	-0.06* (0.03)	13%	-0.17*** (0.03)	62%
Some limitation	-0.19*** (0.03)	42%	-0.19*** (0.03)	69%
Severe limitation	-0.32*** (0.05)	71%	-0.32*** (0.05)	118%
Log of Income	0.45*** (0.02)		0.27*** (0.02)	
Hausman test	*χ*^2^(4):	21.9	*p*-val:	0.00
*Any disability*				
Disability	-0.14*** (0.03)	31%	-0.21*** (0.03)	77%
Log of income	0.45*** (0.02)		0.27*** (0.02)	

Note: Other covariates include: age, gender, ethnicity education level, marital status and employment status of the respondent; household size, housing tenure status, region of residence (rural vs urban), SEIFA quintile, and a time trend.

## Discussion

The results of this study have several implications. Firstly, our results show that with the same level of income, the living standard is lower in households with people with a disability compared to households without members with a disability. This finding indicates a strong relationship between poverty and disability. However, current poverty measures do not take into account disability, therefore, they fail to consider substantial differences in poverty rates between people with and without a disability. Also, the income used in this study included all sources of income, including current disability support, hence it suggests that the current level of government support for people with disabilities is not enough. Secondly, the estimated costs reflected in this study do consider foregone income due to disability. Therefore, policymakers should seriously consider adopting-disability adjusted poverty and inequality measurements. Thirdly, increasing the income (e.g. through government payments) or providing subsidised services for people with a disability may increase the financial satisfaction of these people, leading to an improved living standard. Therefore, policymakers should also consider increased spending for people with a disability. Fourthly, as the National Disability Insurance Scheme (NDIS) only focuses on people with severe disability, to improve the income of people with less severe disabilities, there should be policies addressing job support, workplace support and employability enhancement for people with less restrictive disabilities. Finally, the results of this study can serve as a baseline for the evaluation of the NDIS. Future research replicating our approach after the nationwide rollout of the NDIS in 2019 is needed.

The current study has several limitations. Firstly, for our sensitivity test, we approximated living standards using a dummy variable capturing whether the household can mobilise $2,000-$3,000 from savings. Our understanding is that a different choice of savings level may better represent financial stability; however, we cannot explore this path further due to the data availability. Secondly, we assumed “no limitation” for people who reported having a disability but their response to the severity of their disability question was missing. Therefore, our assumptions may not completely reflect the severity level of theirdisability. Finally, this study did not estimate the cost of disability for different socioeconomic groups despite controlling for ethnicity and socioeconomic status in the regressions. Our estimates also did not identify contributors to income disparities by disability status, which could be investigated using a decomposition approach [18, 19].

## Conclusions

This paper has implicitly estimated the costs of disability in Australia by applying a SoL approach using a large, contemporary, national panel data set. In this paper we were able to: (1) investigate the dynamics of disability and income by using lagged disability and income status, (2) control for unobserved individual heterogeneity and endogeneity of income, and, (3) distinguish between short and long run disability costs using a hybrid panel data regression approach. We found that the average cost of having any disability in the short-run in Australia was 50% of disposable adult-equivalent annual income. This figure varied considerably with the severity of disability, ranging from 19% for people without work-related limitations to 102% for people with severe limitations. Also, the average cost of disability in the long-run was higher at 63% of adult-equivalent disposable income. This was distributed more evenly across severity levels, ranging from 37% for people with no work-related limitations to 94% for people with severe limitations. These results were sensitive to the choice of proxies for standard of living. Highly subjective measures such as overall life satisfaction inflated the cost estimates, and therefore were not recommended. Further, estimates that used cross-sectional data and ignored unobserved individual-specific characteristics (e.g. previous Australian estimates by Saunders [2]) may underestimate the costs of disability.

The results of this study have several implications. Firstly, our results show that with the same level of income, the living standard is lower in households with people with a disability compared to households without members with a disability. This indicates a strong relationship between poverty and disability. However, current poverty measures do not take into account disability, therefore, they fail to consider substantial differences in poverty rates between people with and without a disability. Secondly, the estimated costs reflected in this study do consider forgone income due to disability. Therefore, policy makers should consider adopting disability adjusted poverty and inequality measurements. Thirdly, increasing the income (e.g. through government payments) or providing subsidised services for people with a disability may increase the financial satisfaction of these people, leading to an improved living standard. Thus policy makers need toconsider increased spending for people with a disability. Further, results of this study can serve as a baseline for the evaluation of the National Disability Insurance Scheme (NDIS). Therefore, future research replicating our approach after the nationwide rollout of the NDIS in 2019 is needed.

## Data Availability

The data are publicly available.

## Abbreviations

BHPS: British household panel survey
CIV: Compensating income variation
FRS: Family resources survey
HILDA: Household, income and labour dynamics in Australia survey
NDIS: National disability insurance scheme
OECD: Organization for economic cooperation and development
SDAC: Survey of disability, ageing and carers
SEIFA: Socio-economic index for areas
SoL: Standard of living
